# Loss of post-transcriptional regulation of *DNMT3b* by microRNAs: A possible molecular mechanism for the hypermethylation defect observed in a subset of breast cancer cell lines

**DOI:** 10.3892/ijo.2012.1505

**Published:** 2012-05-31

**Authors:** RUPNINDER SANDHU, ASHLEY G. RIVENBARK, WILLIAM B. COLEMAN

**Affiliations:** 1 Department of Pathology and Laboratory Medicine; 2 Curriculum in Toxicology; 3 Program in Translational Medicine; 4 UNC Lineberger Comprehensive Cancer Center, University of North Carolina School of Medicine, Chapel Hill, NC, USA

**Keywords:** hypermethylator phenotype, DNMT3b, microRNAs, breast cancer

## Abstract

A hypermethylation defect associated with DNMT hyperactivity and DNMT3b overexpression characterizes a subset of breast cancers and breast cancer cell lines. We analyzed breast cancer cell lines for differential expression of regulatory miRs to determine if loss of miR-mediated post-transcriptional regulation of *DNMT3b* represents the molecular mechanism that governs the overexpression of DNMT3b that drives the hypermethylation defect in breast cancer. MicroRNAs (miRs) that regulate (miR-29a, miR-29b, miR-29c, miR-148a, miR-148b) or are predicted (miR-26a, miR-26b, miR-203, miR-222) to regulate *DNMT3b* were examined among 10 hypermethylator and 6 non-hypermethylator breast cancer cell lines. Hypermethylator cell lines express diminished levels of miR-29c, miR-148a, miR-148b, miR-26a, miR-26b, and miR-203 compared to non-hypermethylator cell lines. miR expression patterns correlate inversely with methylation-sensitive gene expression (r=−0.66, p=0.0056) and directly with the methylation status of these genes (r=0.72, p=0.002). To determine the mechanistic role of specific miRs in the dysregulation of *DNMT3b* among breast cancer cell lines, miR levels were modulated by transfection of pre-miR precursors for miR-148b, miR-26b, and miR-29c into hypermethylator cell lines (Hs578T, HCC1937, SUM185) and transfection of antagomirs directed against miR-148b, miR-26b, and miR-29c into non-hypermethylator cell lines (BT20, MDA-MB-415, MDA-MB-468). Antagomir-mediated knock-down of miR-148b, miR-29c, and miR-26b significantly increased *DNMT3b* mRNA in non-hypermethylator cell lines, and re-expression of miR-148b, miR-29c, and miR-26b following transfection of pre-miR precursors significantly reduced *DNMT3b* mRNA in hypermethylator cell lines. These findings strongly suggest that: i) post-transcriptional regulation of *DNMT3*b is combinatorial, ii) diminished expression of regulatory miRs contributes to *DNMT3b* overexpression, iii) re-expression of regulatory miRs reduces *DNMT3b* mRNA levels in hypermethylator breast cancer cell lines, and iv) down-regulation of regulatory miRs increases *DNMT3b* mRNA levels in non-hypermethylator breast cancer cell lines. In conlcusion, the molecular mechanism governing the DNMT3b-mediated hypermethylation defect in breast cancer cell lines involves the loss of post-transcriptional regulation of *DNMT3b* by regulatory miRs.

## Introduction

Aberrant DNA methylation and epigenetic silencing of gene expression are well recognized hallmarks of cancer (1,2), and methylation-dependent gene silencing occurs frequently in breast cancer (3–6). Changes in gene expression patterns secondary to methylation-dependent gene silencing fundamentally contribute to initiation, development, and progression of breast cancer (7–9). We identified a novel hypermethylation defect that is expressed in a subset of breast cancer cell lines and is characterized by epigenetic silencing of methylation-sensitive genes secondary to overexpression of DNMT3b and DNA methyltransferase hyperactivity (10). The hypermethylation defect observed among breast cancer cell lines is associated with a characteristic gene expression signature that reflects methylation-dependent silencing of a panel of epigenetic biomarker genes (including *CDH1, CEACAM6, CST6, ESR1, GNA11, MUC1, MYB, SCNN1A* and *TFF3*) (10). This hypermethylation gene signature was established in human breast cancer cell lines, but also identifies a subset of primary sporadic breast cancers (10). A robust correspondence is observed between the expression of the hypermethylation defect and the basal-like molecular subtype of breast cancer, suggesting that this may be a defining characteristic of this breast cancer subtype (10). However, the molecular mechanism that governs the overexpression of DNMT3b among hypermethylator breast cancer cell lines has not been investigated.

*DNMT3b* is constitutively expressed by all mammalian cell types, but is frequently overexpressed in cancer (11–14). However, unlike other genes that are overexpressed in cancer, the mechanisms accounting for increased *DNMT3b* levels infrequently involve gene mutations and/or gene amplification (15). Likewise, increased *DNMT3b* transcription due to increased *trans*-activation does not commonly occur in cancer (15). Rather, it is now recognized that DNMT3b is subject to post-transcriptional regulation by microRNAs (miRs), which are small non-coding RNAs (19–25 nucleotide long) that regulate gene expression through sequence-specific targeting of mRNAs, producing either translational repression or degradation of the target mRNA (16,17). miRs are expressed in a tissue-specific manner and have been implicated in the regulation of several biological processes, including cellular proliferation, apoptosis, and development (18–21). Altered miR expression is associated with several types of human cancer, including breast cancer (22–25). Recent studies have identified miRs as both regulators of DNA methyltransferase (DNMT) expression and targets of aberrant DNA methylation in various tissue types. The miR-29 family (miR-29a, miR-29b, miR-29c) directly targets DNMT3a and DNMT3b in lung cancer (26) and acute myeloid leukemia (27). Likewise, the miR-148 family (miR-148a, miR-148b) regulates *DNMT3b* in cell lines of multiple origin, including the MCF-7 breast cancer cell line (28). In human bladder cancer, miR-127 is silenced by promoter hypermethylation (29). In similar fashion, miR-148a is epigenetically silenced in human cancer cell lines established from lymph node metastasis from colon, melanoma, and head/neck, suggesting that epigenetic loss of miR-148 is associated with progressive changes such as development of metastatic potential (24). All of these observations indicate direct interactions as well as cross-talk between the DNA methylation machinery and miRs.

In the present study, we analyzed breast cancer cell lines for differential expression of regulatory miRs to determine if loss of miR-mediated post-transcriptional regulation of *DNMT3b* represents the molecular mechanism that governs the overexpression of DNMT3b which drives the hypermethylation defect in breast cancer. The results show that multiple miRs (miR-29c, miR-148a, miR-148b, miR-26a, miR-26b, and miR-203) post-transcriptionally regulate *DNMT3b* in combination and loss of expression of these regulatory miRs contributes to DNMT3b overexpression in hypermethylator cell lines. We also observed that enforced expression of regulatory miRs results in reduced *DNMT3b* mRNA levels in hypermethylator breast cancer cell lines, and that down-regulation of regulatory miRs results in increased *DNMT3b* mRNA levels in non-hypermethylator breast cancer cell lines. These observations combine to suggest that the loss of multiple regulatory miRs that post-transcriptionally regulate DNMT3b levels is involved in the molecular mechanism governing the DNMT3b-mediated hypermethylation defect in breast cancer cell lines.

## Materials and methods

### Cell lines and growth conditions

Human breast cancer cell lines BT20 (ATCC no. HTB19), BT549 (HTB122), Hs578T (HTB126), MCF7 (HTB22), MDA-MB-231 (HTB26), MDA-MB-415 (HTB128), MDA-MB-435S (HTB129), MDA-MB-436 (HTB130), MDA-MB-453 (HTB131), MDA-MB-468 (HTB132), SKBR3 (HTB30), and ZR-75-1 (CRL-1500) were obtained from the Tissue Culture Core Facility of the University of North Carolina Lineberger Comprehensive Cancer Center (Chapel Hill, NC). Human breast cancer cell lines SUM102, SUM149, and SUM185 were a gift from the laboratories of Dr Carolyn I. Sartor (Department of Radiation Oncology, UNC School of Medicine, Chapel Hill, NC) and Dr Stephen Ethier (Department of Pathology, Wayne State University School of Medicine, Detroit, MI). Human breast cancer cell line HCC1937 (CRL-2336) was a gift from the laboratory of Dr William K. Kaufmann (Department of Pathology and Laboratory Medicine, UNC School of Medicine). The normal breast epithelial cell line MCF12A (CRL-10782) was obtained from the ATCC (American Type Culture Collection, http://www.atcc.org/). Cell lines were propagated in growth medium recommended by the ATCC, except for SUM102, SUM149, and SUM185 cells which were cultured in 1:1 mixture of Dulbecco’s modified Eagle’s medium and Ham’s F12 (DMEM/F12, Gibco/Invitrogen Life Technologies, Carlsbad, CA) medium supplemented with 10% horse serum (Gibco/Invitrogen Life Technologies), and 1% antibiotic-antimycotic (Gibco/Invitrogen Life Technologies). Growth medium was refreshed three times weekly unless otherwise specified for antagomir and pre-miR transfections. Cells were maintained at 37°C and 5% CO_2_ (except for MDA-MB-468 which was propagated in 100% atmospheric air).

### RNA extraction for gene expression analysis

Total RNA for gene expression analysis and miR expression analysis was isolated from breast cancer cell lines, MCF12A (normal mammary epithelial cell line), and transfected cell lines (antagomir or pre-miR transfected) utilizing the method of Chomczynski and Sacchi (30), modified for TRIzol Reagent (Invitrogen Life Technologies, Carlsbad, CA), according to the manufacturer’s protocol. Nucleic acid samples were DNAse (Cat no. M610A; Promega, Madison, WI) treated (0.02 U/*μ*l at 37°C for 30 min), and purified using the Qiagen RNeasy mini-kit (Cat no. 74104; Qiagen, Valencia, CA). Isolated RNA was quantified after extraction using a Nanodrop Spectrophotometer (NanoDrop Technologies, Wilmington, DE).

### MicroRNA expression analysis

**Figure 1:**
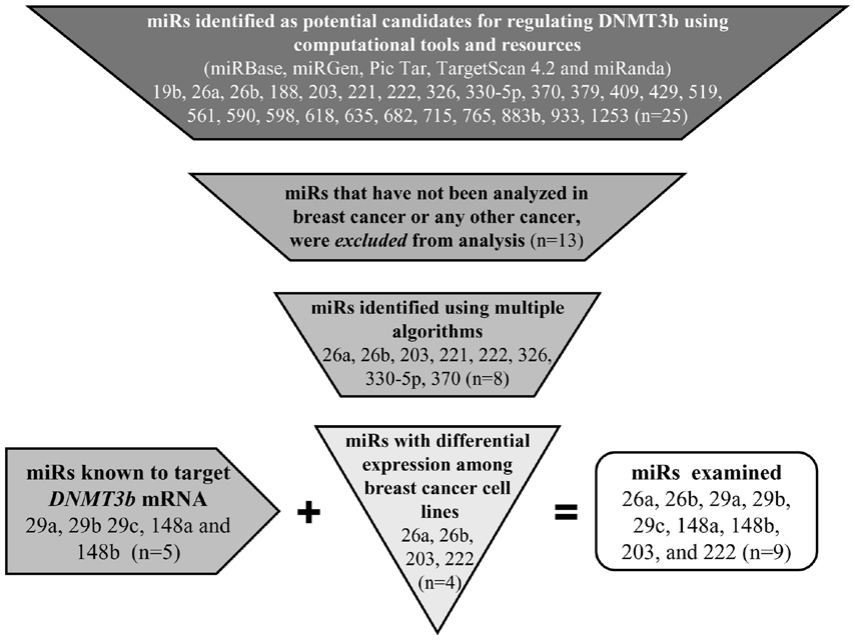
miR regulators of *DNMT3b* mRNA. Schematic illustrating the selection and prioritization of miR regulators of *DNMT3b* for analysis. Several target prediction programs were utilized to predict miR interactions with *DNMT3b*. Criteria for filtering potential candidates are described in the schematic. In addition to selection of candidate miR regulators, known regulators of *DNMT3b* were identified from the literature. This selection strategy yielded nine miRs for examination: miR-29a, miR-29b, miR-29c, miR-148a, miR-148b, miR-26a, miR-26b, miR-203, and miR-222.

*DNMT3b* is subject to miR-mediated post-translational regulation. The miR-29 family (miR-29a, miR-29b, miR-29c) has been implicated in *DNMT3b* dysregulation in lung cancer (26) and acute myeloid leukemia (27), and the miR-148 family (miR-148a, miR-148b) has been implicated in *DNMT3b* dysregulation in cell lines of multiple origins, including the MCF-7 breast cancer cell line (28). We identified candidate miRs as potential regulators of *DNMT3b* using the computational tools of target prediction programs and resources from publicly available databases, including Miranda (http://www.microRNA.org/), TargetScan (http://www.targetscan.org/vert_42/), miRGen (http://www.diana.pcbi.upenn.edu/miRGen/v3/miRGen.html), PicTar (http://pictar.mdc-berlin.de/), and miRBase (http://microrna.sanger.ac.uk/sequences/) computing for target predictions based on searches using Gene symbol DNMT3b (Entrez Gene ID 1789 and Ensembl Gene ID ENSG00000088305). Based on high stringency *in silico* selection criteria that included PicTar score (indicative of HMM maximum likelihood fit), highly conserved miRs, and good mirSVR scores (indicative of seed-site pairing, site context, free-energy, and conservation), we identified 25 additional miRs that potentially target DNMT3b (Fig. 1). We prioritized the candidate miRs based on the available literature and/or their recognition as potential candidates by multiple target prediction programs (Fig. 1). miRs that were differentially expressed among breast cancer cells in primary tumors (23) and cell lines (31) were considered for further analysis. Based upon this computational analysis, we selected nine miRs for examination: miR-29a, miR-29b, miR-29c, miR-148a, miR-148b, miR-26a, miR-26b, miR-203, and miR-222 (Fig. 1).

miR expression analysis was accomplished by real-time PCR utilizing an ABI 7500 real-time PCR System (Applied Biosystems, Foster City, CA) according to TaqMan miRNA assay protocol (Applied Biosystems). Total RNA samples (10 ng) were reverse transcribed using the TaqMan MiRNA Reverse Transcription Kit (Part no. 4366596 Applied Biosystems) and TaqMan miRNA specific primers (Applied Biosystems) according to the manufacturer’s protocol. Real-time primers and probes for miR-29a (Assay ID 000412), miR-29b (Assay ID 000413), miR-29c (Assay ID 000415), miR-148a (Assay ID 000470), miR-148b (Assay ID 000471), miR-26a (Assay ID 000405), miR-26b (Assay ID 000407), miR-203 (Assay ID 000507), miR-222 (Assay ID 002276), and RNU66 (Assay ID 001002) were purchased from Applied Biosystems. These assays specifically detect mature miRNAs (not pre-miRNAs). All real-time PCR reactions were performed in triplicate using TaqMan Universal PCR Master Mix (Cat no. 4324018, Applied Biosystems) in 20 *μ*l volume containing 10 *μ*l TaqMan Universal PCR Master Mix, 1 *μ*l of primers and probe mix of the miR-specific TaqMan MicroRNA Assay (Applied Biosystems), 1.33 *μ*l of RT product, and 7.67 *μ*l of nuclease free water and the following amplification conditions: 95°C for 10 min, 40 cycles of 95°C for 15 sec and 60°C for 1 min. Relative expression levels for each miR were calculated based upon the expression of RNU66 and differences in gene expression were determined relative to MCF-12A using the comparative C_t_ method described in the ABI PRISM 7700 User Bulletin no. 2 (Applied Biosystems).

### Gene expression analysis

Gene expression analysis was accomplished by real-time PCR utilizing an ABI 7500 Real-Time PCR System (Applied Biosystems). Total RNA samples (2 *μ*g) were reverse transcribed using the High Capacity cDNA Reverse Transcription Kit (Part no. 4368814 Applied Biosystems) according to the manufacturer’s protocol. Real-time primers and probes for *CEACAM6* (Hs00366002_m1), *CST6* (Hs00154599_ m1), *DNMT3b* (Hs00171876_m1), *SCNN1A* (Hs00168906_m1), and β-actin (Hs99999903_m1) were purchased from Applied Biosystems. All real-time PCR reactions were performed in triplicate using TaqMan Universal PCR Master Mix (Cat no. 4324018, Applied Biosystems) in 20 *μ*l volume (10 *μ*l TaqMan Universal PCR Master Mix, 1.0 *μ*l TaqMan Real-time primers and probes, and 9 *μ*l cDNA and nuclease-free water) and the following amplification conditions: 95°C for 10 min, 40 cycles of 95°C for 15 sec and 60°C for 1 min. Relative expression levels for each gene were calculated based upon the expression of β-actin for each cell line and differences in gene expression were determined relative to MCF-12A using the comparative C_t_ method described in the ABI Prism 7700 User Bulletin no. 2 (Applied Biosystems).

### DNMT3b protein expression in breast cancer cell lines

Cultured breast cancer cell lines, MCF12A (normal mammary epithelial cell line), and transfected cell lines (antagomir or pre-miR transfected) were lysed in phosphate buffered saline (137 mM NaCl, 2.7 mM KCl, 8 mM Na_2_PO_4_, 2 mM KH_2_PO_4_, pH 7.4) containing 0.1 mM phenylmethanesulphonylfluoride, 1 *μ*g/ml pepstain A, 1 *μ*g/ml leupeptin, 1 *μ*g/ml aprotinin, 1 mM β-glycerol phosphate, 1 mM sodium orthovanadate, and 0.1% Triton X-100. Cell lysates were utilized for western blot analysis using standard methods. Protein concentrations were determined using the Bradford assay (Bio-Rad Quick Start Bradford, Cat no. 500-0205). Protein lysates (20–40 *μ*g) were resolved on 8% SDS-PAGE gels, followed by transfer onto polyvinylidene difluoride (PVDF) membranes (Cat no. 162-0184, Bio-Rad Sequi-Blot PVDF, 0.2 *μ*M pore size, Millipore, Billerica, MA). PVDF membranes were blocked for 30 min in TBST (10 mM Tris-Cl, pH 7.6, 150 nM NaCl, 1% Tween-20) containing 5% milk, and then incubated with either anti-DNMT3b mouse monoclonal antibody (Cat no. IMG-184A Imgenex, San Diego, CA) diluted 1:5000 or anti-actin rabbit polyclonal antibody diluted 1:10,000 (Cat no. sc-1616 Santa Cruz Biotechnology, Santa Cruz, CA) overnight in TBST containing 1% milk. Subsequently, membranes were washed with TBST 3 times for 5 min, and then incubated with a sheep anti-mouse (1:5000, Cat no. NA931 GE Healthcare, Piscataway, NJ) or donkey anti-rabbit (1:10,000, Cat no. NA934 GE Healthcare) horseradish peroxidase-conjugated secondary antibody in TBST containing 1% milk for 1 h at room temperature. The membranes were washed with TBST 3 times for 10 min each, and bound primary antibody was detected using ECL-Plus substrate (GE Healthcare).

### Breast cancer cell line transfection with pre-miRs

Hypermethylator cell lines Hs578T, HCC1937, and SUM185 were selected for pre-miR transfection with miR-148b, miR-26b, and miR-29c. These cell lines exhibit DNMT hyperactivity, express DNMT3b at high levels (10), and exhibit negligible levels of expression of miR-26b, miR-29c, and miR-148b. All pre-miR transfections were performed in triplicate. Pre-miR miRNA precursors (miR-148b, PM10264; miR-26b, PM12899; miR-29c, PM10518) and standard control oligomers were obtained from Applied Biosystems. For optimization purposes, the Pre-miR miRNA Precursor Starter Kit (Applied Biosystems) was utilized for the reverse transfection procedure according to the manufacturer’s protocol using siPORT *NeoFX* Transfection Agent (Part no. AM4510, Applied Biosystems). Four concentrations of transfection reagent (9, 12, 15 and 18 *μ*l) were tested to obtain optimum conditions for pre-miR transfections for each cell line. Transfection reagent was diluted to 300 *μ*l with opti-MEM (Gibco/Invitrogen Life Technologies), incubated for 10 min at room temperature, 24 *μ*l of 6.25 nM of Pre-miR hsa-miR-1 miRNA precursor or Pre-miR negative control no. 1 was diluted to 300 *μ*l with opti-MEM for final concentration of 50 nM and gently mixed with diluted transfection agent before incubating for 10 min at room temperature. The transfection complexes were dispensed into 6-well culture plates, and non-transfected controls were set up in parallel. Cells (2.4×10^5^) were transferred in 2.4 ml of growth medium per well and incubated at recommended growth conditions. After 24 h, the culture medium was replaced with fresh normal growth medium. Two days after transfection, total RNA was extracted from transfected and control cells. The expression level of PTK9 mRNA (target of pre-miR miR-1 miRNA precursor) was assessed by real-time PCR (Hs00702289_s1, Applied Biosystems) according to the manufacturer’s instructions. Optimal transfection was observed with 12 *μ*l transfection reagent in each cell line, producing 75–90% reduction of *PTK9* mRNA after transfection with Pre-miR miR-1. Hs578T, SUM185, and HCC1937 cells were transfected with pre-miR precursors for miR-148b, miR-26b and miR-29c employing the optimized conditions. After 48 h, total RNA was harvested for real-time PCR analysis for miR and gene expression analyses. In addition, transfected and control cells were lysed for western blot analysis (as described above).

### Breast cancer cell line transfection with antagomirs

Non-hypermethylator cell lines BT20, MDA-MB-415, and MDA-MB-468 were selected for antagomir transfection with miR-148b, miR-26b, and miR-29c. These cell lines have lower DNMT activity, express DNMT3b at low levels (10), and exhibit normal levels of expression of miR-26b, miR-29c, and miR-148b. All antagomir transfections were performed in triplicate. Antagomirs (miR-148b, AM10264; miR-26b, AM12899; miR-29c, AM10518) and standard control oligomers were obtained from Applied Biosystems. For optimization of transfection conditions, the reverse transfection procedure was performed using four concentrations of transfection reagent (9, 12, 15 and 18 *μ*l), as described for pre-miR transfections. Transfection reagent was diluted to 300 *μ*l with opti-MEM (Gibco/Invitrogen Life Technologies), incubated for 10 min at room temperature, 24 *μ*l of 6.25 nM of Anti-miR let-7c miRNA inhibitor positive control or Anti-miR negative control no. 1 was diluted to 300 *μ*l with opti-MEM for final concentration of 50 nM and gently mixed with diluted transfection agent before incubating for 10 min at room temperature. Levels of *HMGA2* mRNA (target of Anti-miR let-7c miRNA inhibitor positive control) were assessed by real-time PCR (Hs00171569_m1, Applied Biosystems) after RNA extraction. Optimal transfection was observed with 12 *μ*l transfection reagent in each cell line, producing 1.8-to 2.4-fold increases in HMGA2 mRNA after transfection with Anti-miR let-7c miRNA inhibitor. BT20, MDA-MB-415, and MDA-MB-468 cells were transfected with antagomirs for miR-148b, miR-26b, and miR-29c, and after 48 h, total RNA was harvested for real-time PCR analysis for miR and gene expression analyses. In addition, transfected and control cells were lysed for western blot analysis (as described above).

### Statistical analysis

The values for the mean and standard error of the mean (SEM) were calculated using the statistical function of Microsoft Excel 2007. Statistical significance was determined using an unpaired t-test (two-tailed). Error bars depicted in bar graphs represent SEM of 3–6 independent experiments.

## Results

### Hypermethylator breast cancer cell lines express diminished levels of regulatory miRs

**Figure 2:**
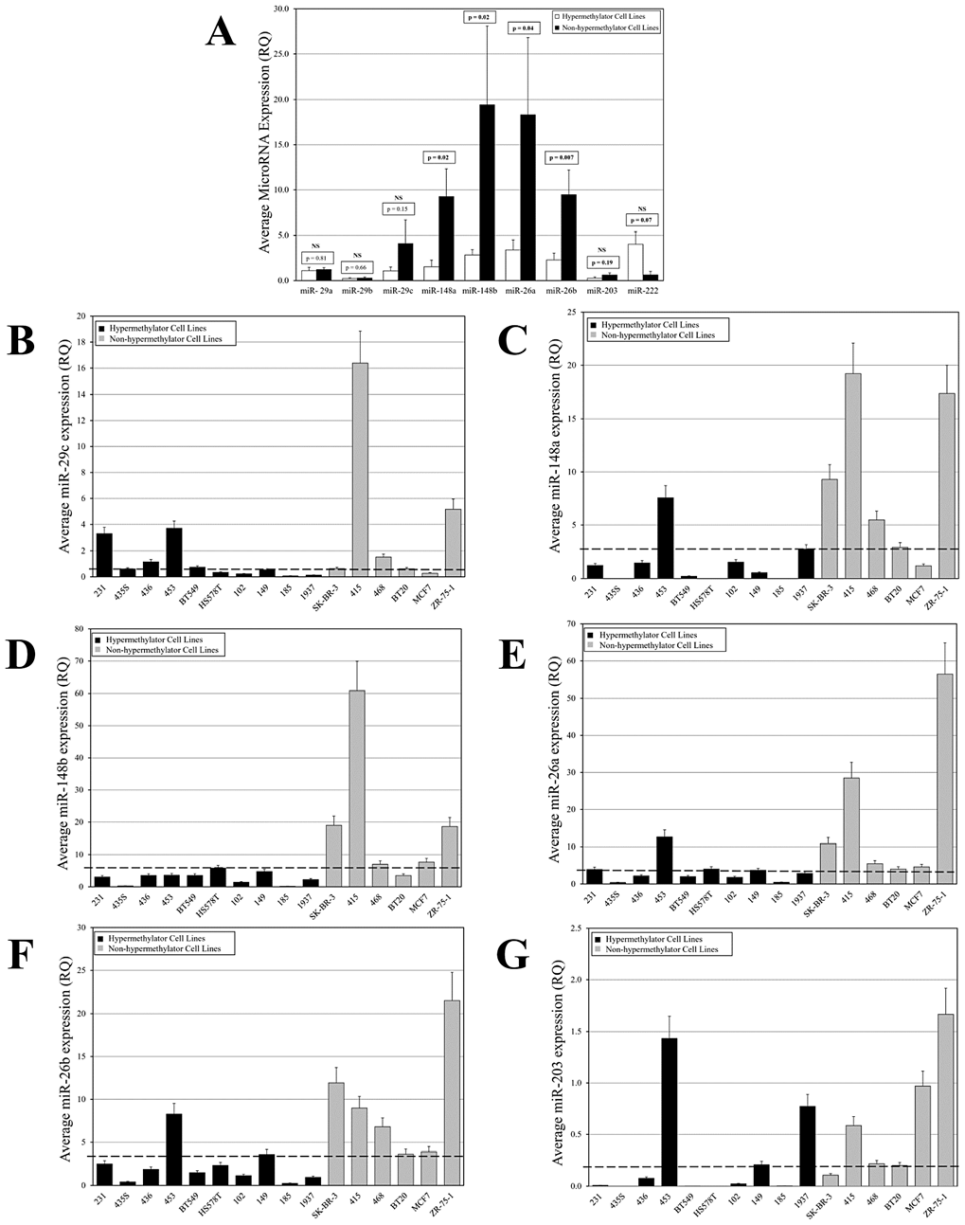
Differential miR expression among hypermethylator and non-hypermethylator breast cancer cell lines. (A) White bars represent average miR expression among hypermethylator cell lines (n=10), and black bars represent average miR expression among non-hypermethylator cell lines (n=6). Comparison of the observed expression levels between hypermethylator cell lines and non-hypermethylator cell lines was accomplished using an unpaired t-test (two-tailed) and corresponding p-values are given (NS, not significant). (B–G) Analysis of miR expression levels among hypermethylator and non-hypermethylator breast cancer cell lines. Hypermethylator cell lines are represented by black bars and non-hypermethylator cell lines are represented by gray bars. The dashed line represents the optimal threshold value determined by Bayesian analysis for correct assignments related to the hypermethylator status of individual cell lines. Each real-time assay was performed in triplicate and error bars represent 15% SEM. MDA-MB-231, MDA-MB-415, MDA-MB-435s, MDA-MB-436, and MDA-MB-453 cell lines are designated 231, 415, 435s, 436, and 453, respectively; SUM102, SUM149, and SUM185 cell lines are represented as 102, 149, and 185, respectively; and HCC1937 is labeled 1937. (B) miR-29c expression, (C) miR-148a expression, (D) miR-148b expression, (E) miR-26a expression, (F) miR-26b expression and (G) miR-203 expression.

Previous investigations identified a hypermethylation defect in a subset of breast cancer cell lines (10). Hypermethylator cell lines display DNMT hyperactivity and overexpression of DNMT3b, in contrast to non-hypermethylator cell lines (10). In the present study, we are investigating possible molecular mechanisms governing DNMT3b overexpression in hypermethylator cell lines, with a focus on miR-mediated regulation of *DNMT3b*. Hence, we examined the levels of expression of select miRs that are known or predicted to regulate *DNMT3b* (miR-26a, miR-26b, miR-29a, miR-29b, miR-29c, miR-148a, miR-148b, miR-203, miR-222) among breast cancer cell lines that differentially express *DNMT3b*. Ten of these cell lines express the hypermethylation defect (BT-549, HS578T, HCC1937, MDA-MB-231, MDA-MB-435S, MDA-MB-436, MDA-MB-453, SUM102, SUM149, SUM185) and six are non-hypermethylators (BT-20, MCF-7, MDA-MB-415, MDA-MB-468, SK-BR-3, ZR-75-1) (10,32; unpublished data). Differential levels of miR expression were observed for six of the nine miRs evaluated, including miR-26a, miR-26b, miR-29c, miR-148a, miR-148b, and miR-203 (Fig. 2A). While there was variability in expression among the miRs examined, in general the hypermethylator cell lines expressed diminished levels compared to the non-hypermethylator cell lines (Fig. 2B–G). miR-29a, miR-29b, and miR-222 did not display the pattern of expression observed with the majority of miRs. miR-29a and miR-29b were expressed at similar levels among breast cancer cell lines irrespective of their methylation status. The lack of differential expression of these miRs is evident from a comparison of average levels in hypermethylator and non-hypermethylator cell lines (Fig. 2A). In contrast to the pattern observed with other miRs, the average expression of miR-222 among hypermethylator cell lines was higher than in non-hypermethylator cell lines. This is consistent with the suggestion that miR-222 functions as an oncogenic miR (33,34).

The average expression of miR-148a, miR-148b, miR-26a, and miR-26b among hypermethylator cell lines was significantly diminished compared to the average expression of these miRs among non-hypermethylator cell lines (p<0.05) (Fig. 2A). Ten/ten (100%) hypermethylator cell lines expressed low levels of miR-148b, and 5/6 (83%) non-hypermethylator cell lines express higher levels of miR-148b (except BT20; Fig. 2D). Likewise, miR-148a is expressed at low levels in 9/10 (90%) hypermethylator cell lines (except MDA-MB-453) and the majority of non-hypermethylator cell lines (5/6, 83%) express miR-148a at higher levels (except MCF7; Fig. 2C). Eight/ten (80%) hypermethylator cell lines display low levels of miR-26a expression (except Hs578T and MDA-MB-453), whereas all non-hypermethylator cell lines (6/6, 100%) express higher levels of miR-26a (Fig. 2E). Similarly, 9/10 (90%) hypermethylator cell lines express low levels of miR-26b (except MDA-MB-453), and 5/6 (83%) non-hypermethylator cell lines express higher levels of miR-26b (except BT20; Fig. 2F). Differences in average expression of miR-29c and miR-203 in hypermethylator cell lines versus non-hypermethylator cell lines were not statistically significant (Fig. 2A), although there was a distinct trend towards lower expression in the hypermethylator cell lines (p=0.15 and p=0.19). Six/ten (60%) of hypermethylator cell lines expressed low levels of miR-29c (except MDA-MB-231, MDA-MB-436, MDA-MB-453, and BT549) and 5/6 (83%) non-hypermethylator cell lines demonstrated higher levels of miR-29c (except MCF7; Fig. 2B). The expression of miR-203 was low in both hypermethylator and non-hypermethylator cell lines, but with differential expression levels (Fig. 2G). Seven/ten (70%) of hypermethylator cell lines expressed miR-203 at low or undetectable levels (except MDA-MB-453, SUM149, and HCC1937), while 5/6 (83%) non-hypermethylator cell lines expressed miR-203 at easily detectable levels (except SK-BR-3).

### Diminished expression of miR-29c, miR-148a, miR-148b, miR-26a, miR-26b, and miR-203 predict hypermethylator status among breast cancer cell lines

We observed differential expression of miR-26a, miR-26b, miR-29c, miR-148a, miR-148b, and miR-203 among breast cancer cell lines with strong trends towards diminished expression in hypermethylators compared to non-hypermethylator cell lines (Fig. 2A). To evaluate the value of individual miR expression levels in the prediction of the methylation status of a given breast cancer cell line, a Bayesian analysis was performed. Threshold values were determined for each of the differentially expressed miRs using correct assignments (CA) as a guiding principle. These threshold values are indicated in Fig. 2B–G. The expression levels of five miRs emerged as excellent individual predictors of methylator status among breast cancer cell lines: miR-148b (CA 94%), miR-26b (CA 94%), miR-148a (CA 88%), miR-26a (88%), and miR-203 (CA 81%). These miRs individually displayed excellent sensitivity (range 80–100%) and specificity (range 83–100%), as well as excellent positive predictive value (PPV range: 89–100%) and negative predictive value (NPV range 71–100%). The best threshold value for miR-29c produced CA 69% (sensitivity, 60%; specificity, 83%; PPV, 86%; and NPV, 56%). The remaining miRs displayed poor predictive value for determination of methylation status of breast cancer cell lines.

### miR expression patterns and miR scores for hypermethylator and non-hypermethylator breast cancer cell lines

**Figure 3:**
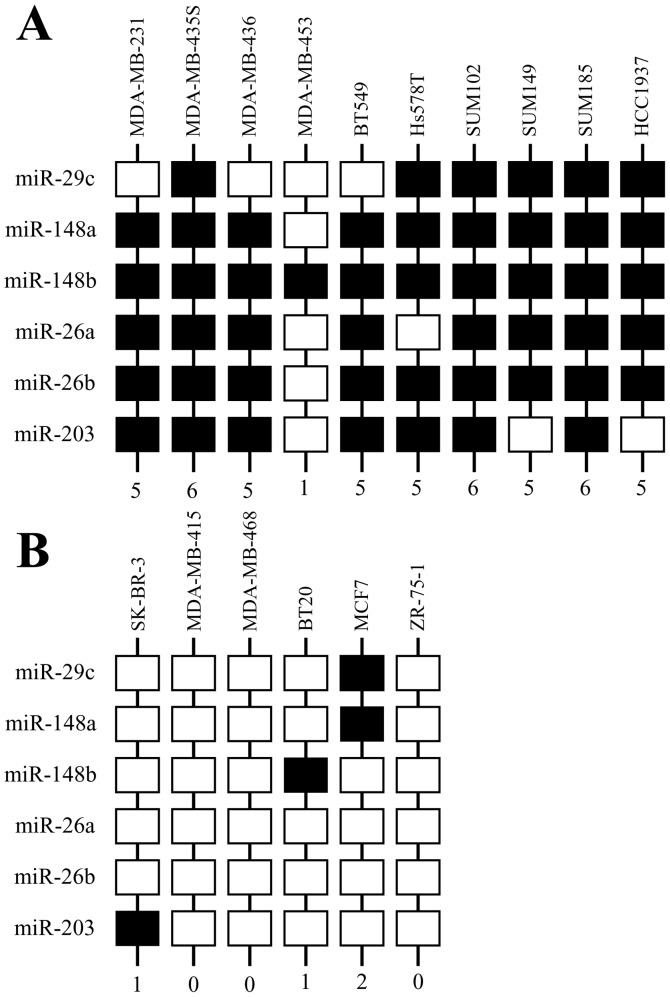
miR expression patterns and miR scores for hypermethylator and non-hypermethylator breast cancer cell lines. Black boxes indicate a measured level of expression for an individual miR that is below the threshold value established through Bayesian analysis, and white boxes indicate a measured level of expression of an individual miR that is above the threshold value established through Bayesian analysis. The numbers at the bottom of each column indicate the miR score, which represents a measure of the number of miRs expressed at diminished levels in an individual cell line. (A) miR expression patterns and miR scores for hypermethylator breast cancer cell lines. (B) miR expression patterns and miR scores for non-hypermethylator breast cancer cell lines.

Six regulatory miRs were chosen for further analysis based on excellent characteristics related to prediction of methylation status (CA, sensitivity, specificity, PPV, and NPV) among hypermethylator and non-hypermethylator breast cancer cell lines, including miR-29c, miR-148a, miR-148b, miR-26a, miR-26b, and miR-203. miR scores were generated for each breast cancer cell line, reflecting the number of miRs with diminished expression. Hypermethylator breast cancer cell lines frequently express diminished levels of this panel of miRs. Nine/ten (90%) hypermethylator cell lines express >5 miRs at diminished levels (Fig. 3A), resulting in higher miR scores. The exception to this is MDA-MB-453, which expresses diminished levels of miR-148b only (Fig. 2D). Hence, MDA-MB-453 has a low miR score reflecting higher levels of expression of the majority of miRs examined (Fig. 3A). Three hypermethylator cell lines (MDA-MB-435s, SUM102, SUM185) express diminished levels of all six miRs examined (Fig. 3A). In contrast to the hypermethylator cell lines, non-hypermethylator cell lines typically express the majority of this panel of miRs at higher levels. Five/six (83%) of non-hypermethylator cell lines express ≥5 miRs at higher levels (Fig. 3B), resulting in lower miR scores. The exception was MCF7, which expresses diminished levels of miR-29c and miR-148a (Fig. 2B and C). Three non-hypermethylator cell lines (MDA-MB-415, MDA-MB-468, ZR-75-1) expressed higher levels of all six miRs in this panel (Fig. 3B). Hypermethylator breast cancer cell lines exhibit an average miR score of 4.9±0.46, whereas, non-hypermethylator cell lines exhibit an average miR score of 0.67±0.33 (p<0.0001).

### miR score correlates with gene expression score and promoter methylation score

**Figure 4:**
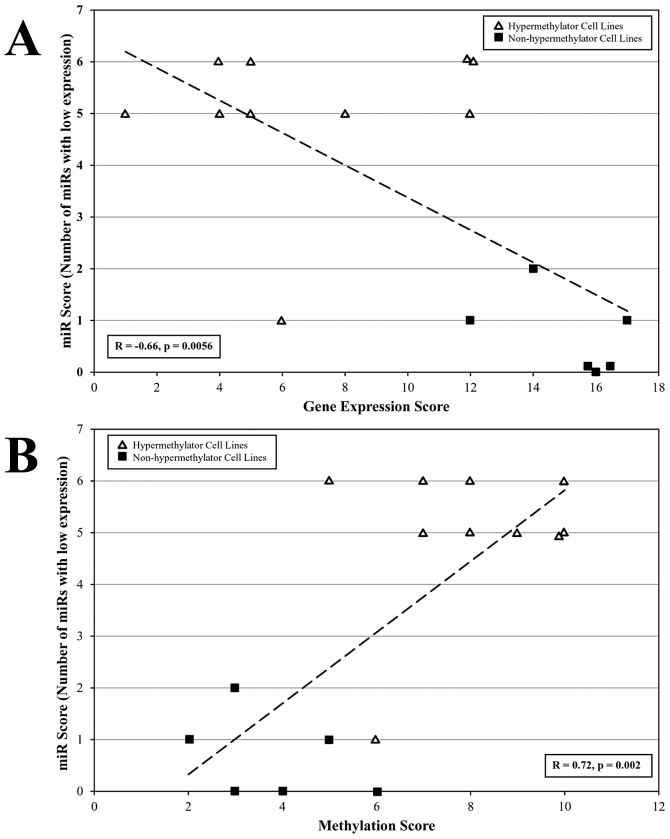
miR expression patterns correlate with methylation-sensitive gene expression status and promoter methylation status among breast cancer cell lines. Correlation of miR expression patterns (miR score) with gene expression levels (based on RT-PCR) and promoter methylation status (based on methylation-sensitive PCR) for methylation-sensitive genes among hypermethylator and non-hypermethylator breast cancer cell lines. Scores were calculated for differentially expressed miRs (miR-29c, miR-148a, miR-148b, miR-26a, miR-26b, and miR-203) and for well-characterized methylation sensitive genes (*CEACAM6, CDH1, CST6, ESR1, GNA11, MUC1, MYB, TFF3*, and *SCNNIA*). Methylation-sensitive gene expression scores and promoter methylation scores were taken from previous studies (10). (A) Relationship between miR score and gene expression score among hypermethylator cell lines (open triangles) and non-hypermethylator cell lines (black squares). (B) Relationship between miR score and promoter methylation status among hypermethylator cell lines (open triangles) and non-hypermethylator cell lines (black squares).

A linear correlation analysis was performed to determine if miR score significantly associates with methylation score and expression score for each breast cancer cell line. Methylation score and expression score reflect the relative promoter methylation status (number of hypermethylated gene promoters) and the relative gene expression status (number of methylation-sensitive genes expressed), respectively, for methylation-sensitive biomarker genes associated with the hypermethylation defect (*CEACAM6, CDH1, CST6, ESR1, GNA11, MUC1, MYB, TFF3* and *SCNNIA*) (10). A strong inverse correlation (r=−0.66, p=0.0056) was observed between miR score and gene expression score (Fig. 4A). Breast cancer cell lines that exhibit diminished expression of multiple regulatory miRs (high miR score) tend to express low levels of methylation-sensitive genes (gene expression score) and cell lines that express higher levels of regulatory miRs (low miR score) tend to express methylation-sensitive genes at higher levels (Fig. 4A). A strong correlation (r=0.72, p=0.002) was observed between miR score and methylation score (Fig. 4B). Breast cancer cell lines that exhibit diminished expression of multiple regulatory miRs (high miR score) exhibit higher methylation scores and cell lines that express higher levels of regulatory miRs (low miR score) tend to have lower methylation scores (Fig. 4B). Previous studies demonstrated significant relationships between overexpression of DNMT3b and gene expression scores and methylation scores for methylation-sensitive genes (10). The current results strongly support the suggestion that loss of miR expression may account for the DNMT3b-mediated hypermethylation defect among breast cancer cell lines that is characterized by methylation-dependent loss expression of methylation-sensitive biomarker genes.

### Co-regulation of miR expression in breast cancer cell lines

**Figure 5:**
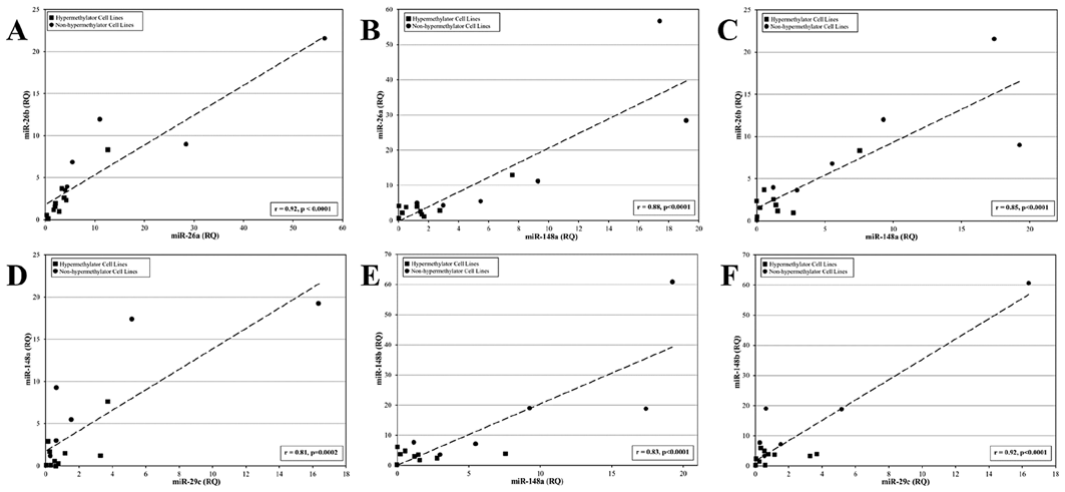
Co-regulation of miR expression among hypermethylator and non-hypermethylator breast cancer cell lines. Hypermethylator cell lines (closed squares) and non-hypermethylator cell lines (closed circles) demonstrate a statistically significant relationship between miR expression levels. The dashed line represents the linear regression trend line (p-values are indicated) for each relationship. (A) Association of expression between miR-26a and miR-26b. (B) Association of expression between miR-148a and miR-26a. (C) Association of expression between miR-148a and miR-26b. (D) Association of expression between miR-29c and miR-148a. (E) Association of expression between miR-148a and miR-148b. (F) Association of expression between miR-29c and miR-148b.

To determine if miRs that regulate DNMT3b are independently regulated or co-regulated at the level of expression, a linear correlation analysis was performed to examine patterns of miR expression among hypermethylator and non-hypermethylator breast cancer cell lines. Statistically significant linear relationships were observed between the levels of expression of several miRs (Fig. 5A–F): miR-26a and miR-26b (r=0.92, p<0.0001), miR-148a and miR-26a (r=0.88, p<0.0001), miR-148a and miR-26b (r=0.85, p<0.0001), miR-29c and miR-148a (r=0.81, p=0.0002), miR-148a and miR-148b (r=0.83, p<0.0001), and miR-29c and miR-148b (r=0.92, p<0.0001). In addition, significant linear relationships were observed for expression of miR-26a and miR-203 (r=0.71, p=0.0019), miR26b and miR-203 (r=0.68, p=0038), miR-26a and miR-29c (r=0.60, p=0.014), miR-148a and miR-203 (r=0.60, p=0.014), and miR-26b and miR-148b (r=0.5, p=0.04). No significant linear relationships were observed for expression of miR-26b and miR-29c, miR-148c and miR-203, or miR-29c and miR-203. Combined, these observations suggest that several miRs that function in the regulation of *DNMT3b* are co-regulated.

### Changes in miR expression levels in hypermethylator and non-hypermethylator breast cancer cell lines after pre-miR and antagomir transfection

**Figure 6:**
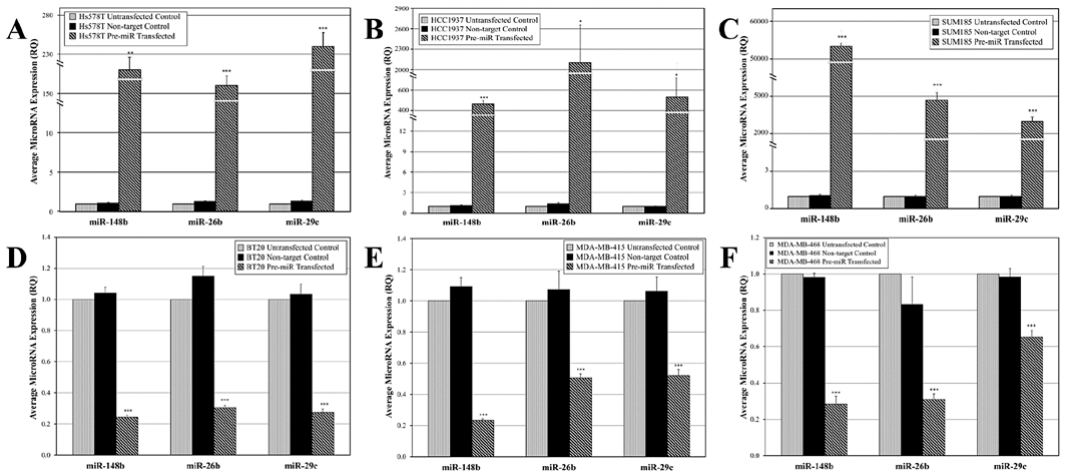
Changes in miR expression levels in hypermethylator and non-hypermethylator breast cancer cell lines after pre-miR and antagomir transfection. Speckled bars represent miR expression levels in untransfected control cells, black bars represent miR expression levels in cells transfected with non-target control oligomers, and crosshatched bars represent miR expression levels in cells after indicated pre-miR or antagomir transfections. (A) Hs578T breast cancer cells re-express miR-148b, miR-26b, and miR-29c after pre-miR transfection. (B) HCC1937 breast cancer cells re-express miR-148b, miR-26b, and miR-29c after pre-miR transfection. (C) SUM185 breast cancer cells re-express miR-148b, miR-26b, and miR-29c after pre-miR transfection. (D) BT20 breast cancer cells express diminished levels of miR-148b, miR-26b, and miR-29c after antagomir transfection. (E) MDA-MB-415 breast cancer cells express reduced levels of miR-148b, miR-26b, and miR-29c after antagomir transfection. (F) MDA-MB-468 breast cancer cells express reduced levels of miR-148b, miR-26b, and miR-29c after antagomir transfection. Each real-time assay was performed 3–6 times and error bars represent SEM. ^*^p<0.05, ^**^p<0.005 and ^***^p<0.0005, compared to untransfected control cells (unpaired t-test).

To determine the mechanistic role of specific miRs in the dysregulation of *DNMT3b* among breast cancer cell lines, the complementary approach of modulating miR levels by transfection of pre-miR precursors (to enforce miR expression in cells lacking a given miR) or transfection of antagomirs (to knockdown miR expression in cells that express normal levels of a given miR) was employed. Transfection of hypermethylator cell lines Hs578T, HCC1937, and SUM185 with pre-miR precursors for miR-148b, miR-26b, and miR-29c resulted in restoration of expression of these miRs (Fig. 6A–C). Following pre-miR transfection, Hs578T cells displayed 210-, 160- and 240-fold increased levels of miR-148b, miR-26b and miR-29c (Fig. 6A). Likewise, pre-miR transfection produced 430-, 2100- and 580-fold increases in miR-148b, miR-26b and miR-29c levels in HCC1937 cells (Fig. 6B), and 54,000-, 4,700-and 2200-fold increases in miR-148b, miR-26b and miR-29c levels in SUM185 cells (Fig. 6C). Non-target control pre-miR precursors did not produce any significant increase in miR-148b, miR-26b and miR-29c levels in any of these cell lines (Fig. 6A–C).

Transfection of non-hypermethylator cell lines BT20, MDA-MB-415, and MDA-MB-468 with antagomirs directed against miR-148b, miR-26b and miR-29c resulted in a significant knockdown of miR-148b, miR-26b and miR-29c levels (Fig. 6D–F). Antagomir transfection of BT20 cells resulted in reduction of miR-148b, miR-26b, and miR-29c levels by 76, 69 and 73%, respectively (Fig. 6D). Likewise, antagomir transfection of MDA-MB-415 cells produced 76, 49 and 48% reductions in miR-148b, miR-26b, and miR-29c levels (Fig. 6E), and antagomir transfection of MDA-MB-468 cells resulted in 72, 69 and 35% reduction in miR-148b, miR-26b and miR-29c levels (Fig. 6F). Non-target control antagomirs did not produce significant alterations in the level of miR-148b, miR-26b and miR-29c in any of these cell lines (Fig. 6D–F).

### Perturbation of regulatory miR expression alters DNMT3b levels in hypermethylator and non-hypermethylator breast cancer cell lines

**Figure 7:**
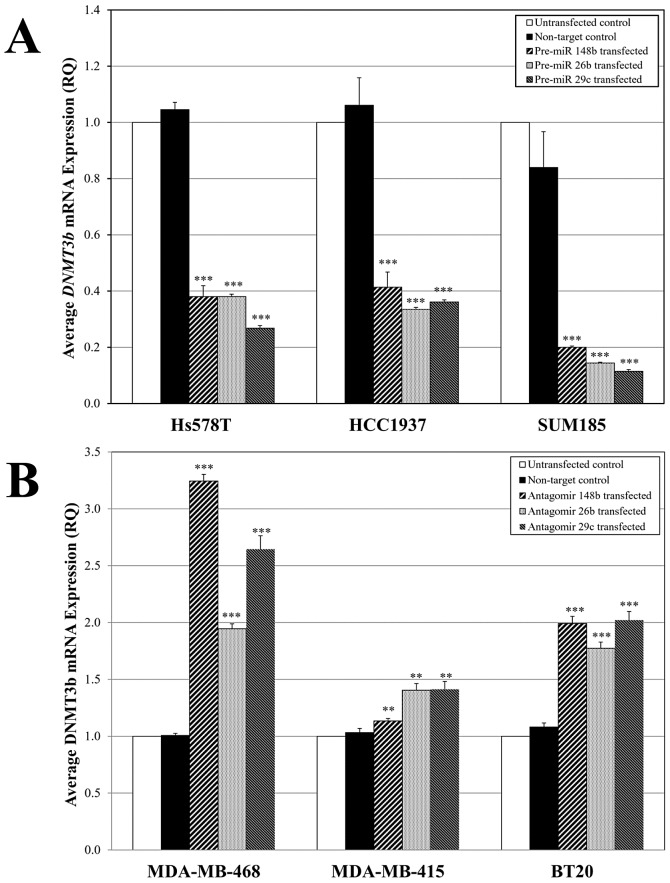
Perturbation of regulatory miR expression affects *DNMT3b* levels in hypermethylator and non-hypermethylator breast cancer cell lines. (A) Hypermethylator breast cancer cells (Hs578T, HCC1937, and SUM185) exhibit significant reduction in *DNMT3b* mRNA levels following pre-miR transfection for miR-148b, miR-26b, and miR-29c. (B) Non-hypermethylator breast cancer cells (MDA-MB-468, MDA-MB-415, and BT20) display significantly increased *DNMT3b* mRNA levels following transfection with antagomirs for miR-148b, miR-26b, and miR-29c. Each real-time assay was performed 3–6 times and error bars represent SEM. ^**^p<0.005, ^***^p<0.0005, compared to untransfected control cells (unpaired t-test).

Enforced expression of miR-148b, miR-26b and miR-29c in hypermethylator cell lines Hs578T, HCC1937 and SUM185 resulted in statistically significant reduction in *DNMT3b* expression levels (Fig. 7A). In Hs578T cells, miR-29c expression reduced *DNMT3b* levels by 73%, and expression of miR-148b and miR-26b produced 62% reduction in *DNMT3b* levels (Fig. 7A). Similar results were obtained in HCC1937 cells with 58–64% reductions of DNMT3b levels in response to enforced expression of miR-148b, miR-26b and miR-29c (Fig. 7A). The most dramatic effect of enforced pre-miR expression on *DNMT3b* levels was observed in SUM185 cells. Expression of miR-29c in SUM185 cells resulted in an 88% decrease in *DNMT3b* mRNA (Fig. 7A). Likewise, expression of miR-148b and miR-26b in SUM185 cells produced 80 and 82% reduction in *DNMT3b* levels (Fig. 7A). Transfection of non-target control pre-miR precursors did not produce any significant change in *DNMT3b* levels in Hs578T, HCC1937 and SUM185 cells (Fig. 7A). Western blot analysis of cell lysates from Hs578T, HCC1937 and SUM185 cells following pre-miR transfection failed to detect significant alterations in DNMT3b protein levels (data not shown). However, the failure to detect changes in DNMT3b protein is likely due to the transient (48 h) nature of this assay system and the relatively long half-life of the DNMT3b protein. With persistent (stable) miR re-expression, we expect to see decreases in DNMT3b. Likewise, assessment of methylation-sensitive gene expression (for *CEACAM6, CST6* and *SCNN1A*) in Hs578T cells after enforced expression of miR-148b, miR-26b and miR-29c did not reveal changes in levels of expression compared to control cells (data not shown), consistent with the lack of change in DNMT3b protein levels. As above, with persistent (stable) miR re-expression, we expect to see alterations in methylation-sensitive gene expression coordinate with changes in DNMT3b levels.

Antagomir-mediated knockdown of miR-148b, miR-26b and miR-29c in non-hypermethylator cell lines MDA-MB-468, MDA-MB-415 and BT20 resulted in statistically significant increases in *DNMT3b* expression levels (Fig. 7B). The most dramatic effects were observed in MDA-MB-468 cells, where miR-148b knockdown produced a 3.2-fold increase in *DNMT3b* mRNA, whereas knockdown of miR-26b and miR-29c resulted in 2-and 2.6-fold increases in *DNMT3b* levels, respectively (Fig. 7B). Comparable increases in *DNMT3b* expression levels (1.8-to 2-fold) were observed in BT20 cells following knockdown of miR-148b, miR-26b and miR-29c (Fig. 7B). More modest increases of *DNMT3b* levels (1.2-to 1.4-fold) were observed in MDA-MB-415 cells after knockdown of miR-148b, miR-26b and miR-29c, but these alterations were statistically significant. Transfection of non-target control antagomirs did not produce any significant change in *DNMT3b* levels in these cell lines (Fig. 7B). Similar to the results obtained with pre-miR-transfected hypermethylator cell lines, western blot analysis of cell lysates from MDA-MB-468, MDA-MB-415, and BT20 cells following antagomir transfection failed to detect significant alterations in DNMT3b protein levels (data not shown). Failure to detect changes in DNMT3b protein is likely due to the transient (48 hour) nature of this assay system. With persistent (stable) miR knockdown, we expect to see increased DNMT3b levels. Further, assessment of methylation-sensitive gene expression (for *CEACAM6, CST6* and *SCNN1A*) in MDA-MB-468 cells after antagomir-mediated knockdown of miR-148b, miR-26b and miR-29c did not reveal changes in levels of expression compared to control cells (data not shown), consistent with the lack of change in DNMT3b protein levels in this short-term assay system. As above, with persistent (stable) miR knockdown, we expect to see alterations in methylation-sensitive gene expression coordinate with changes in the level of DNMT3b.

## Discussion

Epigenetic changes significantly contribute to the normal regulation of gene expression and when dysregulated can significantly contribute to carcinogenesis (35,36). Aberrant epigenetic silencing of tumor suppressor genes and other negative mediators of cell proliferation has been documented in the development and progression of breast cancer (3,5,9). The CpG island methylator phenotype (or CIMP) represents a major epigenetic mechanism of colorectal carcinogenesis that has also been recognized in cancers affecting other tissues (37–39). We have identified a hypermethylation defect in a subset of human breast cancer cell lines and primary breast cancers that are characterized by DNMT hyperactivity, overexpression of DNMT3b, and concurrent methylation-dependent silencing of numerous genes (including *CDH1, CEACAM6, CST6, ESR1, GNA11, MYB, MUC1, SCNN1A* and *TFF*) (10). Mining of microarray-based expression data identified a strong cluster of primary breast cancers that display a gene expression signature associated with hypermethylation defect (10). A strong association was established between the expression of the hypermethylation defect signature and the basal-like molecular subtype of breast cancers (10). Basal-like breast cancers are typically classified as triple-negative, reflecting lack expression of estrogen and progesterone receptors (ER^−^/PR^−^), and absence of HER2 gene amplification (HER2^−^) (40,41). Hence, patients with basal-like breast cancer are not responsive to targeted therapies like tamoxifen (targeting ER) and trastuzumab (targeting HER2) (42,43). The poor prognosis associated with basal-like breast cancer and lack of druggable targets makes the fundamental observation of the co-segregation of the hypermethylation defect with basal-like breast cancer to be of utmost significance. Our observations suggest strongly that the DNA methylation machinery (and specifically DNMT3b) represents new/novel molecular target for development of drugs and treatment strategies for basal-like breast cancer.

In the present study, our goal was to elucidate the molecular mechanism accounting for overexpression of DNMT3b in hypermethylator breast cancer cell lines. Recent studies link miRs to the post-transcriptional regulation *DNMT3b* expression in various tissues. Loss of expression of members of miR-29 family and overexpression of *DNMT3b* has been shown in lung cancer (26) and acute myeloid leukemia (27). Likewise, there is evidence supporting the negative regulation of *DNMT3b* by miR-148a and miR-148b in cell lines of multiple origins (28). The results of the present study strongly suggest that loss of regulatory miR expression contributes to *DNMT3b* overexpression in hypermethylator breast cancer cell lines. This evidence includes: i) differential expression of regulatory miRs between hypermethylator and non-hypermethylator cell lines, ii) significantly diminished expression of miR-29c, miR-148a, miR-148b, miR-26a, miR-26b, and miR-203 among hypermethylator breast cancer cell lines, iii) pre-miR-mediated re-expression of miR-148b, miR-26b, or miR-29c in hypermethylator breast cancer cell lines (Hs578T, HCC1937 and SUM185) reduces DNMT3b mRNA levels, and iv) antagomir-mediated knockdown of miR-148b, miR-26b, or miR-29c in non-hypermethylator breast cancer cell lines (MDA-MB-468, MDA-MB-415, and BT20) leads to increased *DNMT3b* mRNA levels. The observed loss of regulatory miRs in expression of the pro-cancerogenic hypermethylation defect suggests that these miRs possess a tumor suppressor-like function in breast, similar to other tissues (26–28).

miRs are predicted to post-transcriptionally regulate more that 60% of all protein-encoding genes in mammals and contribute to almost every cellular process, normal and pathological (44). miRs have been recently been established as key players in carcinogenesis, with functions that can be oncogenic or tumor suppressor-like (15). Our results suggest loss of combinations of miR-29c, miR-148a, miR-148b, miR-26a, miR-26b and miR-203 is associated with expression of the hypermethylation defect in breast cancer cell lines, consistent with the idea that these miRs function as negative mediators of the neoplastic phenotype. Diminished levels of these miRs have been documented in various forms of cancer, supporting the suggestion that these miRs possess tumor suppressor-like function. Reduced expression of miR-26a occurs in hepatocellular carcinoma, oral squamous cell carcinoma, bladder cancer, thyroid anaplastic carcinoma, Burkitt’s lymphoma, acute myeloid leukemia, papillary carcinoma, prostate cancer, and breast cancer (44–46). miR-26b expression is diminished in Hodgkin’s lymphoma, oral squamous cell carcinoma, and prostate cancers (46). miR-29c expression is depressed in nasopharyngeal carcinomas, bladder tumors, chronic lymphocytic leukemia, acute myeloid leukemia, lung cancers, cholangiocarcinoma, esophageal squamous cell carcinoma, and pancreatic ductal adenocarcinoma (44–48). miR-148a is down-regulated in breast cancers, papillary thyroid carcinoma, pancreatic ductal adenocarcinoma, prostate cancer, and colorectal adenocarcinoma (45,46). miR-148b is expressed at reduced levels in oral squamous cell carcinoma, papillary thyroid carcinoma, prostate cancer, colorectal adenocarcinoma, and pancreatic ductal adenocarcinoma (46). miR-203 levels are diminished in oral squamous cell carcinoma, chronic myeloid leukemia, hepatocellular adenomas, and esophageal squamous cell carcinoma (45,46). These studies from the literature document loss or diminished expression of miR-29c, miR-148a, miR-148b, miR-26a, miR-26b, and miR-203 in various forms of cancer, including breast in some cases.

Several molecular mechanisms contribute to miR dysregulation in cancer, including genetic abnormalities (such as chromosomal rearrangement, deletion, amplification, or sequence mutations) and epigenetic changes (methylation-dependent silencing of miR expression or alterations in the miRNA biogenesis machinery) (44). Numerous miR genes (more than 50%) are positioned within or close to chromosomal fragile sites and other genomic regions associated with cancer (44). Genetic alterations involving these chromosomal regions result in dramatic alteration of miR expression levels (44). Likewise, numerous studies report promoter hypermethylation as an important mechanism leading to loss of miR expression in cancer (15). Loss of miR-203 expression is associated with fragile site on chromosome, 14q32 (49), as well as through promoter hypermethylation in hematopoietic malignancies (49,50). miR-148a and miR148b are also susceptible to methylation-dependent silencing in cancer (15). We found miR-203 to be significantly co-regulated with miR148a and miR-148b, suggesting the possibility of a common epigenetic mechanism accounting for their diminished expression in hypermethylator cell lines. These examples from the literature suggest that loss of regulatory miR expression leading to *DNMT3b* dysregulation could be the result of genetic or epigenetic mechanisms. Given the linkage between basal-like breast cancers and expression of the hypermethylation defect, loss of regulatory miR expression leading to *DNMT3b* overexpression may represent a very early and significant molecular alteration during the natural history of breast carcinogenesis. Further, loss of regulatory miR expression and establishment of the hypermethylation defect (with *DNMT3b* overexpression) may determine and/or drive the basal-like molecular subtype of breast cancer.

The purpose of this study was to elucidate the molecular mechanism governing the overexpression of DNMT3b associated with the expression of hypermethylation defect in breast cancer. The results of this study strongly suggest that multiple miRs post-transcriptionally regulate *DNMT3b* in combination and that loss of expression of these regulatory miRs contributes to *DNMT3b* overexpression. Mechanistic dissection of the role of selected miRs in the regulation of *DNMT3b* among hypermethylator and non-hypermethylator breast cancer cell lines produced additional evidence for the importance of this molecular regulating network in determination of the methylation status of breast cancer cell lines. Re-expression of regulatory miRs reduces *DNMT3b* mRNA levels in hypermethylator breast cancer cell lines, and down-regulation of regulatory miRs increases *DNMT3b* mRNA levels in non-hypermethylator breast cancer cell lines.

In conclusion, the molecular mechanism governing the DNMT3b-mediated hypermethylation defect in breast cancer cell lines involves the loss of post-transcriptional regulation of *DNMT3b* by regulatory miRs.

## Abbreviations:

CDH1: E-cadherin
CEACAM6: carcinoembryonic antigen-related cell adhesion molecule 6
CIMP: CpG island methylator phenotype
CST6: cystatin E/M
DNMT3a: DNA methyltransferase 3a
DNMT3b: DNA methyltransferase 3b
ESR1: estrogen receptor 1
GNA11: guanine nucleotide binding protein, alpha 11
HER2: human epidermal growth factor receptor 2
HMGA2: high mobility group AT-HOOK 2
miR: microRNA
MUC1: mucin 1
MYB: v-myb myeloblastosis viral oncogene homolog
PR: progesterone receptor
PTK9: protein tyrosine kinase 9
SCNN1A: sodium channel nonvoltage-gated 1 alpha
TFF3: trefoil factor 3
